# Contrast-Enhanced Ultrasound (CEUS) for Echographic Detection of Hepato Cellular Carcinoma in Cirrhotic Patients Previously Treated with Multiple Techniques: Comparison of Conventional US, Spiral CT and 3-Dimensional CEUS with Navigator Technique (3DNav CEUS)

**DOI:** 10.3390/cancers3021763

**Published:** 2011-03-30

**Authors:** Francesco Giangregorio

**Affiliations:** Department of Gastroenterology, Guglielmo da Saliceto Hospital, Via Taverna 49, Piacenza 29121, Italy; E-Mail: f.giangregorio@alice.it; Tel.: +39-339-827-3840; Fax: +39-052-330-2057

**Keywords:** hepatocellular carcinoma, liver, detection, SonoVue, three dimensional ultrasound, contrast enhanced ultrasound, spiral CT

## Abstract

A commercially available technique named “NAVIGATOR” (Esaote, Italy) easily enables a 3-D reconstruction of a single 2-D acquisition of Contrast Enhanced Ultrasound (CEUS) imaging of the whole liver (with a volumetric correction thanks to the electromagnetic device of NAVIGATOR). Aim of the study was to evaluate this “panoramic” technique in comparison with conventional US and spiral CT in the detection of new hepatic lesions. 144 cirrhotic patients (previously treated for hepato cellular carcinoma (HCC)) in follow-up with detection of 98 new nodules (N), 28 multinodular (Nmulti), 14 loco-regional regrowth (LR) 94 efficaciously treated without new nodules (neg) and four multinodular without new nodules, were submitted to 200 examinations with this new technique from November 2008 to November 2009. 3DNavCEUS was performed using SonoVue (Bracco), as contrast agent, and a machine (Technos MPX, Esaote). Spiral CT and 3DNav CEUS were performed in the same month during follow up. Sens.,Spec.,diagn.-Acc.,PPV and NPV were evaluated; comparison and differences between the techniques were obtained with chi-square (SPSS release-15). Final diagnosis was: 98 new lesions (N) (one to three), 28 multinodular HCC (Nmulti) and 14 loco-regional regrowth (LR); in 94 no more lesions were observed during follow-up; conventional US obtained: 58 N (+18 multinodularN and 8 LR), 40 false negative (+10 Nmulti and 6 LR) (sens:59.2, spec:100%, Diagn Accur:73.6, PPV:100; NPV:70.1); spiral CT obtained: 84N (+26-multinodularN and 14-LR), 14 false-negative (+2-Nmulti), and one false-positive (sens:85.7, spec:97.9%, Diagn Accur:90.9, PPV:97.7; NPV:86.8); 3DNAV obtained: 92N (+28 multinodularN and 14LR), 6 false-negative, and two false-positives (sens:93.9, spec:97.9%, Diagn Accur:95.6, PPV:97.9; NPV:93.9). 3-DNav CEUS is significantly better than US and almost similar to spiral CT for detection of new HCC. This technique, in particular, showed the presence of lesions even in the cases not detected with spiral CT.

## 1. Introduction

The fine detection, the exact spatial position, and the characterization of thin lesions in the liver, are the goals of modern radiological imaging [1] in cirrhotic [2] and cancer [3,4] patients. Recent technological improvements, in particular with Magnetic Resonance Imaging (MRI), have led to spatial reconstruction of liver [2–4].

The importance of three-dimensional imaging is accepted for several reasons: the exact location of a given lesion, its relations to neighboring structures and organs (a lesion's continuity may be assessed, particularly in infiltrating neoplastic growths), an accurate measurement of volume in expanding lesions and hence therapy planning, which is particularly relevant for liver tumors undergoing alcoholization, injection of antitumor agents, or therapy with radiofrequency waves, shock waves, *etc.*, as well as response monitoring [5].

Spiral CT [6] and MRI [2–4] are used for this purpose: They are contrast-enhanced imaging techniques (able to show the arterial, portal and late phase), high temporal resolution techniques (able to capture images in a short acquisition time), high spatial resolution techniques (able to use thin layers between two-dimensional (2D) imaging sections, which contribute to reduce volume artifacts), panoramic techniques (able to reconstruct the whole liver in a breath-hold period) [2,3].

Three dimensional Ultrasonography (3-D US) is a technique able to rebuild a consecutive bi-dimensional US scanning in a three dimensional space. In fact, It acquires a series of 2D images and forms a volume data set, which can be manipulated interactively to display the region of interest from arbitrary orientations and can provide direct 3D images [7–10].

Our previous, as yet unpublished, experience [11] showed that 3-D US was a technique useful to perform detection of HCC: We demonstrated that it was possible to detect HCC using the 3-D software in the US machine (TECHNOS MPX, Esaote, Italy) with free-hand technique. The limit was that the 3-D reconstruction of the liver was dependent on the type of scanning; in other words, the acquired volume was not corrected in the three dimensional space. To obviate this problem, we used an adapted software within a commercially available machine, called NAVIGATOR (Esaote, Italy) (and recently described as real-time guidance using a fusion imaging system that combines US and CT information in the targeting and subsequent radiofrequency thermal ablation (RFTA) ablation of a liver target inconspicuous [12]), to capture a continuous scanning of the entire liver. This scanning, called NAVIGATOR 3-D CEUS, was possible both with conventional US and with contrast-enhanced US; the post-processing fusion technique consistently demonstrated that the CT and US 3-D reconstruction of the liver were exactly superimposable.

Aim of our study was to use contrast-enhanced US to detect HCC during follow-up in cirrhotic patients with HCC previously treated with non-surgical techniques (such as percutaneous ethanol injection (PEI), radiofrequency thermal ablation (RFTA), trans arterial chemoembolization (TACE)) in comparison with spiral CT in monitoring treated HCC (with multiple techniques) and in the detection of new hepatic lesions.

## 2. Materials and Methods

### 2.1. Patients

144 cirrhotic patients with HCC (previously treated with non-surgical treatments) were consecutively enrolled from November 2008 to November 2009: 76 males (mean age ± sd: 71.4 ± 10.5 years) and 68 females (75 ± 10 years). All patients were Hepatitis C Virus (HCV)-positive. 116 patients were in Child A class during these 12 months follow-up (64 in Child A5, 36 in Child A6), while 28 in Child B (16 in Child B7, 8 in Child B8 and 4 in Child B9). According to Barcelona-Clínic Liver Cancer (BCLC) classification [13], 70 patients were “early” and 74 “intermediate”.

At the time of first treatment, 128 patients were in Child A (Child A5:112; Child A6: 16) and 16 in Child B (Child B7: 12; child B: 4).

#### 2.1.1. Previous treatments

Eight patients were treated with RFTA and two patients also with surgical resection (these two patients received previous loco-regional therapies in other liver segment (6th for the first patient; 4th for the second) and the last treatment was surgical because new HCC were superficial (both on 5th segment, near to gallbladder not treatable with RFTA/PEI) at least 12 months before the 3-D NAV CEUS; 134 patients were treated in the previous six months with different techniques: 54 with RFTA (34 early and 20 intermediate), 48 with PEI (20 early and 28 intermediate) and 32 with TACE (12 early and 20 intermediate).

Follow up was performed with conventional US every three months, with spiral CT and 3-D NAV CEUS every six months; during follow up 98 new lesions (N) (20 single, 12 double, 18 triple), 28 multinodular HCC (Nmulti) and 14 loco-regional regrowth (LR) were detected in 50 patients; all lesions were inferior to three centimeters; in 94 no more lesions were observed during follow-up.

#### 2.1.2. Methods

3DNav CEUS was performed using a suspension of sulfur hexafluoride in sterile saline (SonoVue, Bracco, Italy), as contrast agent. It is characterized by the combination of improved stability with favorable resonance behavior at low acoustic pressure. This allows minimally disruptive contrast specific imaging at low mechanical index (MI) and enables effective investigations over several minutes with the visualization of the dynamic enhancement pattern in real time [14].

Every examination was performed with 2.4 mL of SonoVue with a low mechanical index (40 Kpascal).

An adapted software inside a commercially available machine, called NAVIGATOR (Esaote, Italy), was used to capture a continuous scanning of the entire liver.

**Figure 1.**
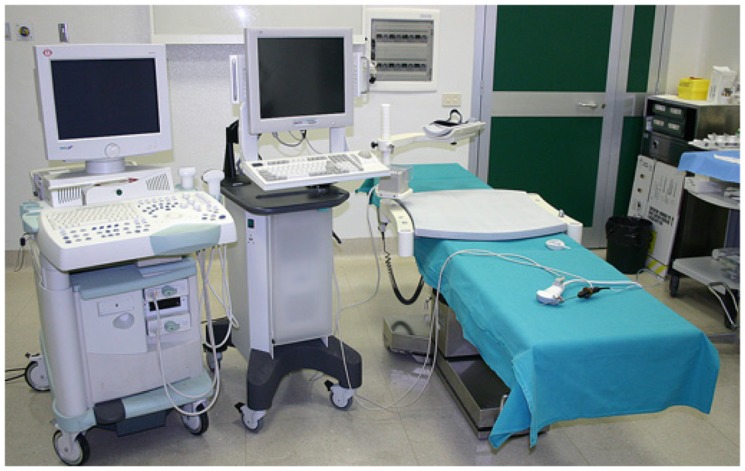
Image of navigator system: From left to right: Ultrasound machine, TECHNOS MPX, NAVIGATOR SYSTEM (with touch screen monitor) connected with the “navigation system”, composed by a passive electro-mechanical sensor (near to the patient) and the “active” one (attached to the ultrasound probe).

**Figure 2.**
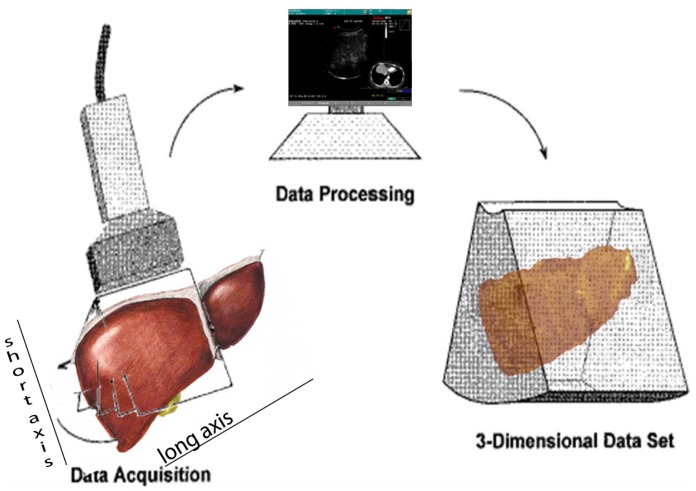
Draft of 3-D reconstruction: LEFT: Bidimensional continuous scanning of the liver evaluating its short axis through its long axis; the planes captured along the liver are rendered in a 3-dimensional reconstruction using software within the Navigator System.

Navigator (Figure 1) is composed of a computer with a touch screen monitor, Tracking system type PCIBirds (Ascension technology-Degrees of freedom: Six (position and orientation); Translation range, any direction: Standard transmitter 0–30 (76.2 cm); Angular range: All attitude; Static accuracy standard sensor: 0.040 (1.0 mm) RMS position 0.15 degree RMS orientation). The navigation system is coupled with a US machine (Technos MPX; Esaote, Genoa, Italy). PCI bird is formed by an active sensor, united to the US probe, and a passive sensor, near to the patient. The active sensor transmits its spatial position and movements in comparison to the passive one; the probe movements are registered and the 3-D software inside Navigator system is able to correct the static 3-D reconstruction with the US scanning in comparison to probe movements. The final result is an accurate spatial reconstruction of the volume independent of the type of 2-D acquisition (Figure 2).

**Figure 3.**
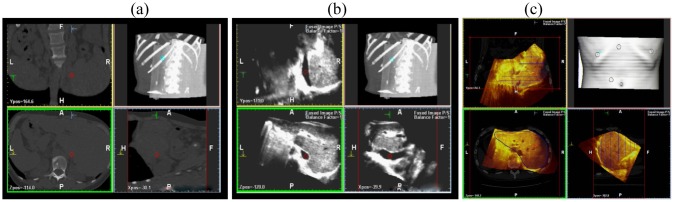
3-D reconstruction of bidimensional spiral CT sequences (**a**); bidimensional US sequences (**b**); the fusion technique allows to superimpose the two volumes, showing a perfect overlapping (**c**).

This scanning was possible both with conventional US and with contrast-enhanced US; the navigator system is furthermore able to perform a volumetric reconstruction of liver with CT scanning and to compare the two different volumes (US and CT) employing the fusion technique (Figure 3).

**Figure 4.**
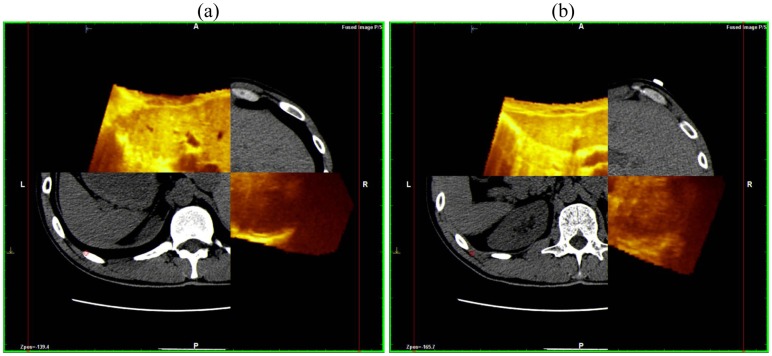
Fusion technique with NAVIGATOR system: Two different planes of reconstruction ((**a**) and (**b**)) of CT and US perfectly overlap. This system is able to separate the two planes (yellow for US, grey for CT) showing the perfect alignment of the margins with each other.

This superimposes each resliced layer of US and CT volumes showing both on the monitor: The post-processing fusion technique has consistently demonstrated that the CT and US 3-D reconstruction of the liver were exactly superimposable (Figure 4).

**Figure 5.**
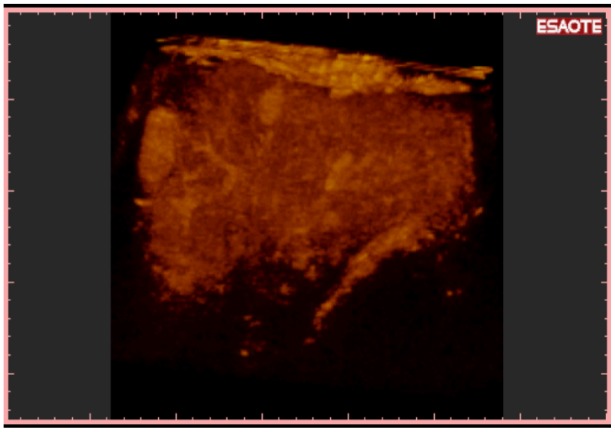
3-D NAV CEUS reconstruction with parenchymal rendered volume: Two new HCC in different liver segments are evident. This kind of reconstruction is able to show HCC nodules inside liver parenchyma; this is performed offline with the software (inside TECHNOS MPX) specific for 3-D rendering.

**Figure 6.**
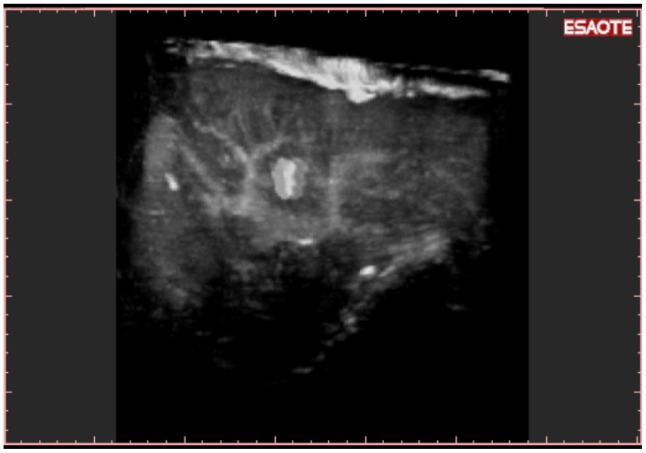
3-D NAV CEUS reconstruction with vascular rendered volume of the same acquisition as in Figure 5. The software can “translate” the volumetric data in different kinds of visualization. In Figure 5, the new HCC are inserted in the parenchimal volume, while here the main vascularization of liver is appreciable in “one shot vision”.

To easily perform a 3-D reconstruction of a 2-D acquisition of CEUS imaging of the whole liver, the 2-D acquisition is performed with real time scanning perpendicularly on the long-axis of the liver, for a complete 2-D image of its short-axis (Figure 2). The subsequent reconstructions of these planes show a rendered volume with a “parenchimal” aspect (Figure 5) or with a “vascular” map (Figure 6) of all the hepatic segments with an acquisition in the early arterial and in portal phase.

3-D NAV CEUS was performed on the same days as the spiral CT/MRI. Follow up was performed with Alfa Feto Protein (AFP) evaluation and conventional US every three months and with spiral CT and 3-D NAV CEUS every six months. In case of AFP growth and/or US suspect for local recurrence or new HCC, 3-D NAV CEUS was repeated after conventional US: For this reason during six months follow-up 3 3-D NAV CEUS were performed in four patients, two examinations in 20 patients and only one examination in 48 patients; 200 examinations with this new technique from November 2008 to November 2009 were performed.

Spiral CT was considered the “imaging” gold standard technique; MRI was performed in 11 cases, spiral CT and 3-D NAV CEUS were discarded.

Sensitivity, specificity, diagnostic accuracy, positive predictive value (PPV) and negative predictive values (NPV) were evaluated; comparison and differences between the techniques were obtained with chi-square (SPSS release 15).

## 3. Results and Discussion

During follow-up, detection of new nodules was performed: 98 new HCC (N) (20 single, 12 double and 18 triple), 28 multinodular (Nmulti), 14 loco-regional regrowth (LR); no new detection was performed in 94 patients efficaciously treated (neg) and in four multinodular patients (Multineg). Results are summarized in Table 1; Conventional US was able to detect 8/14 (57.1%) local recurrences, 58/98 (59.1%) new nodules in other segments and 18/28 (64.2%) new cases of multinodular HCC (eight less than 2 cm, two more than 2 cm). Six false negative local recurrences were all less than a 2 cm site in segments II, IV and VIII. 40 false negative for new nodules had these characteristics: Dimensions less than 2 cm in 34 HCC, between 20 and 25 mm in six nodules. Segmental distribution was the following: 12 in segment VIII, eight in segment IV, six in segment V, four in segment II and the other 10 (multinodular) in more segments.

Spiral CT correctly detected all the local recurrences, 84/98 (85.7%) new nodules in other segments and 26/28 (92.8%) multinodular HCC (two cases of small active nodules, suspected only by AFP elevated values and diagnosed only by MRI). Dimensions of 14 false negative for CT were: 12 nodules less than 2 cm, two 3 cm; their segmental distribution was: 8 nodules in segment VIII and two nodules in segment IV, VI and VII (eight nodules were not detected in new segments in comparison to HCC previously treated and six in the same segment). In two cases, spiral CT erroneously diagnosed an artero-venous malformation as new HCC (false positive) (confirmed by MRI and follow-up).

**Figure 7.**
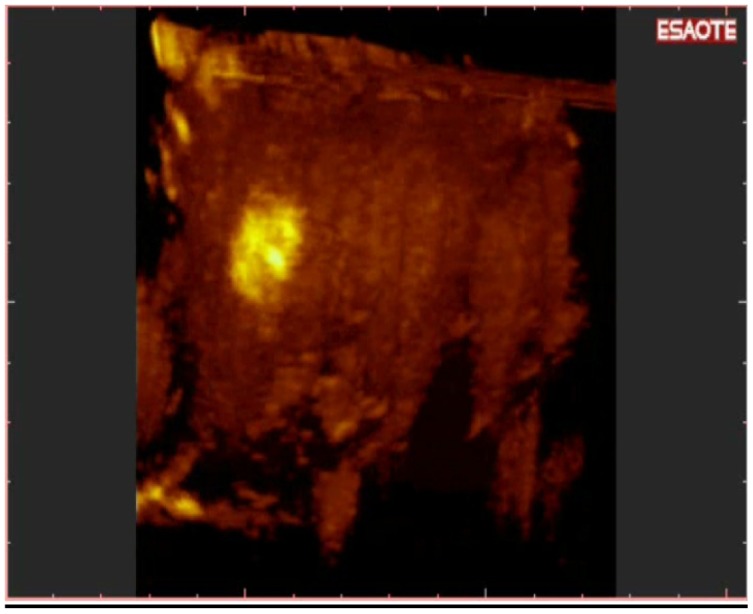
Detection of new HCC: A new HCC was detected during follow-up: 3-D NAV CEUS was able to show the exact position of the nodule in tridimensional space.

**Figure 8.**
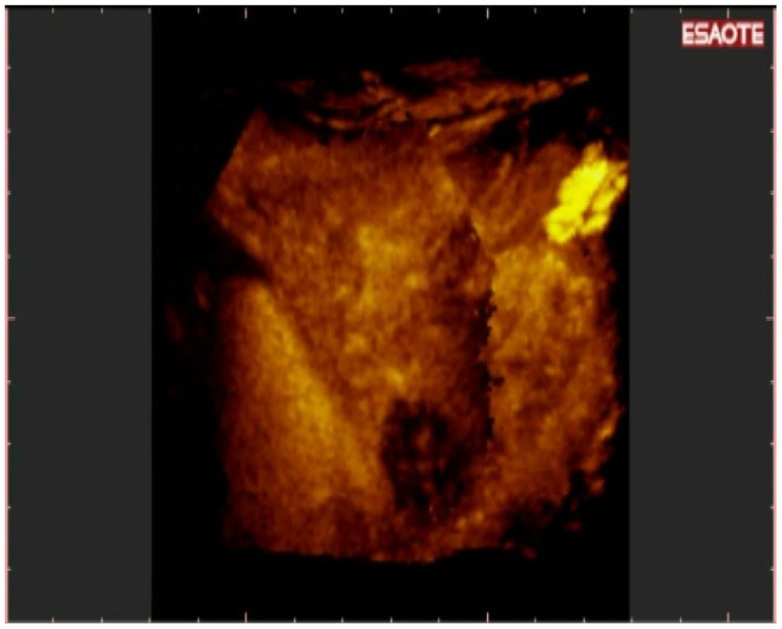
The liver volume can be studied “plane by plane” with reslicing technique, able to show a previously treated HCC of the VII segment in the same patient.

**Figure 9.**
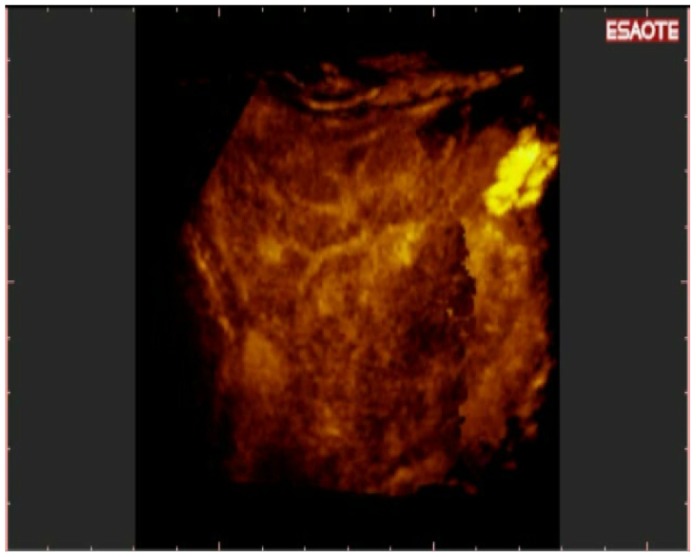
New multinodular HCC of the IV segment. These nodules were not visible with conventional US. 3-D NAV CEUS was performed due to the appearance of elevated values of AFP.

3-D NAV CEUS correctly detected all the local recurrences, 92/98 (93.87%) of new nodules (Figure 7) and 26/28 (92.8%) multinodular HCC (Figure 8 and Figure 9) (the same patient with small multinodular HCC false negative also for spiral CT). Dimensions of eight false negative for 3-D NAV CEUS were: Six nodules less than 2 cm, two 3 cm; their segmental distribution was: Four nodules in segment IV and four nodules in segment VI. In two cases 3-D NAV CEUS erroneously diagnosed an artero-venous malformation as new HCC (false positive) (confirmed by MRI and follow-up).

**Figure 10.**
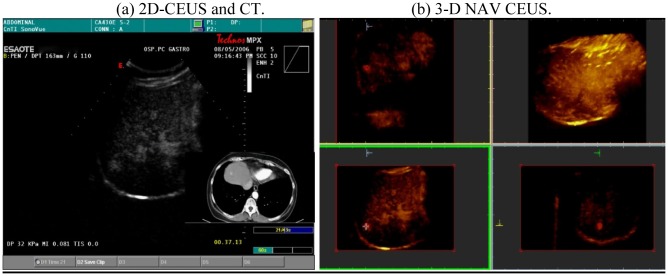
(**a**). Bidimensional acquisition plane of a female patient with elevated values of AFP and conventional US negative for new nodules. Only spiral CT was able to detect a small HCC in VIII segment. 3-D NAV CEUS detected the nodule; (**b**). The 3-D reconstruction easily showed the single nodule on the three different planes (red cross) and on the volumetric reconstruction. After the PEI treatment, AFP became normal and no new nodules were detected during the six months follow up.

40 new single nodules were evaluable only by spiral CT and not by conventional US: 34/40 (85%) were detected by 3-DNAV CEUS (Figure 10).

Conventional US sensitivity was only equal to 59.2%, but with high specificity (100%). Overall diagnostic accuracy was 73.6%. Spiral CT had a relatively low sensitivity (85.7%) and low diagnostic accuracy in comparison to 3-D NAV CEUS sensitivity (93.87%) and diagnostic accuracy (95.6%). Differences between conventional US and 3-D NAV CEUS were statistically significant (p < 0.001). Instead, differences between spiral CT and 3-D NAV CEUS were not statistically significant (p = ns). Results are summarized in Table 2.

## 4. Discussion

Usually, the detection of HCC in cirrhotic patients is performed by conventional US and by CT, according to EASL [15] and AASLD guidelines [16]. 3-D NAV CEUS technique, described here, may be used instead of simple conventional US and for safe spiral CT execution during follow up in cirrhotic patients with previously treated HCC. This particular population was chosen to have a more high pre-test probability of new HCC detection; in other words, this system was tested in a population with a higher probability of new HCC. Of course, this system will be useful (and may replace conventional US) also for screening of cirrhotic patients, but no studies are available at the moment.

3-D US is a relatively new technique for which accuracy in volume measurements in liver was demonstrated [7]. It is a not real time reconstruction, but it is able to reveal a three dimensional space in a similar manner to CT and RMs [7]. For these characteristics, 3-D US [9,17] or 4-D US [18,19] (that means real-time 3-D US) were also described as efficacious techniques in ultrasonographically guided biopsy of hepatic [9,17,18] or superficial [19] masses. Furthermore, three-dimensional sonography was useful in delineation of expandable radio frequency electrodes, improvement of operator confidence level, determination of applicator placement, and visualization of the position relationship between the applicator and adjacent critical structures during procedures of liver cancer ablation under image guidance [20].

Recently, 3-D CEUS was employed in liver lesions characterization [21,22] and in the post-treatment evaluation of effectiveness of loco-regional therapies [23,24]. 3-D CEUS was also employed in combination with navigation system to characterize active HCC and to monitor post-procedural effectiveness of local ablative therapies [24,25].

3-D US had three big disadvantages: It has no contrast-enhanced imaging, nor panoramic technique, and is operator dependent.

New US systems are able to perform 3-D reconstruction using contrast-agents imaging. This means that CEUS 3-D reconstruction is based on the macro and microcirculation. In this way, it enables detecting the enhanced vasculature of HCC during arterial phase and the typical late wash-out in the same manner as performed by spiral CT/MRI.

However, the three-dimensional reconstruction of images may be rather more complex for ultrasonography, since the various 2D slices are manually obtained—rather than automated—using conventional ultrasound, and hence image acquisition depends on the explorer's skills. In addition, the number of slices that may be obtained is incalculable with no established start or end, since one more slice is always feasible. Finally, the acquired volume has to be “corrected” in relation to the type of scanning.

To solve the problem of the “panoramicity” of US scan (it was impossible to scan all the liver in a few seconds during arterial phase), we thought of performing single acquisitions of the liver parenchyma by visualizing its “short” axis: the superimposition of these images, taken by scanning the liver through the “long” axis, made the acquisition of all the liver parenchyma during arterial phase possible, in a manner similar to spiral CT one.

To correct US reconstructions of the liver in the three-dimensional space, new fusion-(US and CT or MRI) and navigation-systems for US were developed: They are a real-time guidance using a fusion imaging system that combines ultrasound (US) and computed tomography (CT) in the targeting and subsequent radiofrequency (RF) ablation of a liver target inconspicuous on US. Ohto and colleagues [26] showed that contrast-enhanced 3D fusion ultrasonography enables the display of images combining the plane shift and opacity control modes to show tumor vessels, including minute vascular flow within hepatic tumors, in a 3D perspective and to identify tumor-specific vascular flow patterns.

Hirooka [27] first demonstrated that Virtual US (a system that generates Three-dimensional (3D) images using thin sections from multi-detector row computed tomography and computer software, simulating images obtained using conventional ultrasonography) has proven useful for visualization of HCC nodules that cannot be seen under conventional US. Furthermore, Virtual US (VUS) was a useful tool for US-guided treatment of HCC. The same author confirmed this experience *in vitro* and *in vivo* this experience [28]. The same group, ultimately, used the CEUS with VUS to assess the therapeutic response to RFA of HCC. Moreover, the number of spiral CT scans required was reduced by this approach [29].

Chopra and co-workers developed a navigation system for tumor resection (Optoelectronic navigation with section mode visualization in two orthogonal planes) in soft tissue based on 3D ultrasound imaging and optical tracking [30]. This system was validated [31] and employed [32] for accurate ultrasound-based navigation of resections of liver tumors.

Recently, Crocetti and colleagues tested the “NAVIGATOR system”, (the one employed in our experience to correct the three dimensional reconstruction in the space) concluding that this is both feasible and accurate [12]. With the navigator system, our group was able to reconstruct the whole liver during the 15–20 seconds of the typical arterial phase [14] with a single scanning of liver along its long axis; ideally, this is the same system as spiral CT, that performs a continuous scanning of the liver, with the difference that CT scans the liver along its short axis (direction from the head to foot), while US probe scanning.

We demonstrated that the 3-D NAV CEUS, connected to navigation system, is able to detect and to characterize new HCC in a similar manner to spiral CT. This is the first experience demonstrating that CEUS is able to detect HCC during follow up in cirrhotic patients, even though CEUS detection of HCC is actually considered difficult because full surveillance of the whole liver is impossible [14].

## 5. Conclusions

3-D NAV CEUS was able to detect and to characterize HCC, in a manner similar to CT, in a population of cirrhotic patients with previously treated HCC during follow up. This system may reduce or sometimes substitute spiral CT controls during follow up in this category of patient.

## Figures and Tables

**Table 1.** Cases of detection of HCC during follow up for conventional US, spiral CT and 3-D NAV CEUS. Differences between conventional US and 3-D NAV CEUS were statistically significant (p < 0.001). However, differences between spiral CT and 3-D NAV CEUS were not statistically significant (p = ns).

	**True + ve**	**True** − **ve**	**False + ve**	**False** − **ve**
**Conventional US**	58 N (18 NMulti + 8LR)	94 (+4Multineg)	0	**40 (10 NMulti + 6 LR)**
**3DNav CEUS**	92N (+26 Nmulti + 7LR)	94 (+2Multineg)	2	**6(+2 Nmulti)**
**Spiral CT**	84N (+26Nmulti + 14LR)	92 (+4Multineg)	2	**14 (+2 Nmulti)**
**Final diagnosis**	**98N (+14Nmulti + 7LR)**	**94 (+2Multineg)**	**-**	**-**

**Table 2.** Sensitivity, specificity, diagnostic accuracy, positive and negative predictive values of conventional US, 3-D NAV CEUS and spiral CT.

**CONVENTIONAL US**	59.2	100	73.6	100	**70.1**
**3DNav CEUS**	93.9	97.9	95.6	97.9	**93.9**
**Spiral CT**	85.7	97.9	90.9	97.7	**86.8**
